# Costs of *Neisseria meningitidis* Group A Disease and Economic Impact of Vaccination in Burkina Faso

**DOI:** 10.1093/cid/civ600

**Published:** 2015-11-09

**Authors:** Anaïs Colombini, Caroline Trotter, Yvette Madrid, Andromachi Karachaliou, Marie-Pierre Preziosi

**Affiliations:** 1 Independent Consultant, World Health Organization Initiative for Vaccine Research, Geneva, Switzerland; 2 Disease Dynamics Unit, Department of Veterinary Medicine, University of Cambridge, United Kingdom; 3 Independent Consultant; 4 Meningitis Vaccine Project, Department of Immunization, Vaccines and Biologicals, World Health Organization, Geneva, Switzerland; 5 Meningitis Vaccine Project, PATH, Ferney-Voltaire, France

**Keywords:** meningitis, case management, vaccination, economic impact, Burkina Faso

## Abstract

***Background.*** Five years since the successful introduction of MenAfriVac in a mass vaccination campaign targeting 1- to 29-year-olds in Burkina Faso, consideration must be given to the optimal strategies for sustaining population protection. This study aims to estimate the economic impact of a range of vaccination strategies in Burkina Faso.

***Methods.*** We performed a cost-of-illness study, comparing different vaccination scenarios in terms of costs to both households and health systems over a 26-year time horizon. These scenarios are (1) reactive vaccination campaign (baseline comparator); (2) preventive vaccination campaign; (3) routine immunization at 9 months; and (4) a combination of routine and an initial catchup campaign of children under 5. Costs were estimated from a literature review, which included unpublished programmatic documents and peer-reviewed publications. The future disease burden for each vaccination strategy was predicted using a dynamic transmission model of group A *Neisseria meningitidis*.

***Results.*** From 2010 to 2014, the total costs associated with the preventive campaign targeting 1- to 29-year-olds with MenAfriVac were similar to the estimated costs of the reactive vaccination strategy (approximately 10 million US dollars [USD]). Between 2015 and 2035, routine immunization with or without a catch-up campaign of 1- to 4-year-olds is cost saving compared with the reactive strategy, both with and without discounting costs and cases. Most of the savings are accrued from lower costs of case management and household costs resulting from a lower burden of disease. After the initial investment in the preventive strategy, 1 USD invested in the routine strategy saves an additional 1.3 USD compared to the reactive strategy.

***Conclusions.*** Prevention strategies using MenAfriVac will be significantly cost saving in Burkina Faso, both for the health system and for households, compared with the reactive strategy. This will protect households from catastrophic expenditures and increase the development capacity of the population.

Burkina Faso is one of the few countries whose boundaries lie wholly within the African “meningitis belt,” and thus experiences a particularly high incidence of meningococcal meningitis, with epidemics occurring regularly [1, 2]. In 2010, Burkina Faso successfully implemented a nationwide preventive campaign with a new conjugate vaccine, known as MenAfriVac, against *Neisseria meningitidis* group A (MenA) [3, 4]. There have been no confirmed cases due to MenA in Burkina Faso since 2010 [2–5], and a substantial overall reduction in the meningitis burden [6], although group W remains a threat [7]. To sustain population-level protection against MenA following the 2010 introductory campaign, the country plans to incorporate MenAfriVac into the routine infant immunization schedule with 1 dose at the age of 9 months in late 2015 or early 2016 together with a single campaign among cohorts of children born since the campaign. This follows the recommendation made by the Strategic Advisory Group of Experts (SAGE) of the World Health Organization (WHO) in October 2014, which advised that meningitis belt countries should introduce MenAfriVac into the routine childhood immunization program within 5 years of campaign completion, together with a one-time catch-up campaign for young children born since the initial mass vaccination who would be outside the age window when the routine immunization program starts [8].

Prior to MenAfriVac introduction in 2010, the public health response to MenA relied on the detection of localized epidemics through surveillance and subsequent reactive immunization campaigns with polysaccharide vaccines [9] (as it still does for disease due to other meningococcal serogroups [10]). The effectiveness of this strategy is limited, largely because when vaccination campaigns are implemented, the epidemic may already be beyond its peak [11, 12]. Because this strategy does not prevent cases and epidemics from occurring, health systems can be severely disrupted and costs to the affected households can amount to one-third of the annual gross domestic product (GDP) per capita in Burkina Faso [13, 14].

This study aims to estimate the costs and savings of alternative preventive immunization strategies with MenAfriVac in Burkina Faso over a 26-year time period, compared with a reactive vaccination strategy.

## METHODS

### General Methodology

We estimate the economic impact of different MenA vaccination strategies in Burkina Faso, in terms of costs and savings for the health system and households and, as such, take a societal perspective. The study is a cost-of-illness study. By definition, cost-of-illness studies measure the economic burden of a disease and estimate the maximum amount that could be saved by reducing that burden [15].

We consider a 26-year time period, from 2010 until 2035, with 2010 being the year of the preventive campaign targeting 1- to 29-year-olds in Burkina Faso and 2015 the anticipated year of the introduction of routine MenAfriVac immunization. The study is both retrospective (real costs from 2010 to 2014) and prospective from 2015 onward, where future costs are projected. New cases are included during the entire study period. We therefore differentiate the time horizon into 2 periods: 2010–2014 and 2015–2035. The 2035 cutoff is based on an expected 10-year duration of protection of MenAfriVac against MenA and an expected waning of the effects of the 2010 campaign around 15 years after vaccine introduction, in the absence of further immunization [16].

The study compares the costs of reactive campaigns for individuals aged 2–30 years with a polysaccharide A + C meningococcal vaccine (reactive strategy) to each of the 3 new vaccination strategies aimed at preventing MenA using MenAfriVac while targeting different age groups : (1) a single preventive campaign in 2010 for individuals aged 1–29 years (preventive strategy); (2) routine immunization at 9 months of age 5 years after the preventive campaign (routine strategy); and (3) routine immunization at 9 months of age 5 years after the preventive campaign, and a catch-up of children born since the preventive campaign (combination strategy).

The incidence of MenA in each of the vaccination strategies is predicted from a transmission dynamic model of MenA and MenAfriVac immunization [16]. The model is designed to capture the typical epidemiology of MenA in the meningitis belt, with periodic but irregular epidemics occurring in the dry season. This model estimates the number of cases occurring per year on a national level and does not predict localized epidemics. Vaccination with MenAfriVac is implemented according to the strategies above, and influences disease epidemiology through effectiveness against disease and carriage (90% in both cases), thus providing direct and indirect protection. In the model, MenAfriVac coverage is assumed to be 95% for the 2010 campaign and 80% for routine and subsequent catch-up. The number of cases of MenA is estimated by applying the annual incidence rates shown in Table 1 to the population of Burkina Faso [17].

**Table 1.** Yearly Incidence Rate of Group A *Neisseria meningitidis* in Burkina Faso, 2011–2035

Year	Incidence Rate of MenA (per 100 000)		
	Preventive Campaign	Routine 1 Dose	Routine + Catch-up
1	0	0	0
2	0	0	0
3	0	0	0
4	0	0	0
5	0	0	0
6	0	0	0
7	0	0	0
8	0	0	0
9	0	0	0
10	0	0	0
11	0	0	0
12	0.0001	0	0
13	0.0022	0	0
14	0.075	0	0
15	1.4	0	0
16	14.1	0.00017	0
17	40.98	0.013	0
18	73.9	0.14	0
19	75.6	0.32	0
20	52.4	0.93	0.0003
21	34.17	3.41	0.038
22	14.19	4.26	0.35
23	5.49	9.59	0.93
24	7.89	16.6	3.17
25	6.85	15.7	5.9

The considered incidence of MenA in the absence of any preventive strategy is 24.7 per 100 000.

Abbreviation: MenA, *Neisseria meningitidis* group A.

Costs and cases are primarily undiscounted, but we also explored the sensitivity of results to 2 alternative discounting scenarios: discounting of 3% for both cases and costs and discounting of costs at 3% with no discounting of cases. All costs were adjusted to 2012 US dollars (USD). For the period 2015–2035, the price of vaccines is an average of the expected price during that period.

### Cost Calculation

Different types of costs are calculated: (1) the costs of case management and of vaccination for the health system, and (2) the direct nonmedical costs (DNMCs) and indirect costs (ICs) for households. We assume that case management of meningitis is free for households and is all supported by the health system, according to the policy promoted by the international community during epidemics of meningitis in Burkina Faso [9]. We further assume that in households there is no self-medication nor visit to a traditional healer prior to contact with a health center or hospital. Both these assumptions are conservative. Costs of sequelae have not been taken into account.

#### Health System Costs

The case management costs are calculated by multiplying the estimated number of cases of MenA under each strategy by an average cost of case management calculated using a multilinear regression analysis including data from 27 countries on the direct medical costs (DMCs) of meningitis cases in low- and middle-income countries [18]. Original data for Burkina Faso come from Colombini et al [13].

The costs of reactive campaigns are based on the total costs of reactive vaccination estimated in a study performed in Burkina Faso in 2007 [14], divided by the number of cases in that study period to give an average cost per case. The average cost per case is then multiplied by the estimated incidence of cases of MenA under a reactive strategy between 2010 and 2035. In reality, reactive vaccination only occurs when an epidemic threshold is reached, so only cases that occur in areas that reach an epidemic threshold will yield a reactive vaccination cost. As the model does not predict the occurrence of localized outbreaks, it is not possible to mirror the true policy.

As an alternative, we estimated the cost of reactive vaccination based on the number of affected districts in Burkina Faso that were immunized in the last MenA epidemic in 2006–2007. Here we assumed that a similar epidemic would occur every 10 years, requiring reactive campaigns in 46 districts with 1 district per year requiring a reactive campaign in interepidemic years. The median district population size in Burkina Faso in 2010 was 233 315, and we assumed that 75% of the district population would be targeted (as a proxy for those aged 2–30 years) at a cost of 1.45 USD per person vaccinated [14].

The cost of the preventive campaign was derived from the WHO report on the 2010 preventive campaign in Burkina Faso and Gavi financial documents and includes total vaccine costs and total delivery costs. The costs of vaccination of the other strategies is based on (1) the unit delivery costs derived from a review of national program documents; (2) the projection of fully loaded price for vaccines and injection supplies per dose; and (3) an estimate of the number of doses required for each strategy given the target population estimated from United Nations population data [17]. The average delivery cost per dose of the catch-up campaign is considered to be the same as for the preventive campaign. The average delivery cost per dose of routine vaccination is derived from the data available on the comprehensive multiyear plan of the immunization program in Burkina Faso [19], which consists of the sum of the specific costs of all the activities needed to administer routine vaccines, divided by the total number of vaccine doses supplied (see Table 2 for more details). We assume that there are no fixed costs and that the cost of immunization is shared proportionally to the number of doses.

**Table 2.** Elements of the Methodology: Mean Costs Used in the Study and Other Methodological Points on Cost Calculation

	Cost Parameters^a^	Sources of Data	Methodological Notes
All Strategies	Mean Cost per Case		
Case management costs for the health system	50.73	Portnoy et al [18] Colombini et al [13]	Field study in Burkina Faso during the epidemic season (2006–2007) Cost includes prepositioning and distribution of medicines during epidemics, district laboratory analyses for case diagnosis and choice of treatment, patient care Multiple regression analysis (Portnoy et al [18])
DMCs for households	…		Hypothesis (conservative): Medical care of meningitis is free of charge for households; all case management costs are captured at the health system level Households do not seek informal care
DNMCs for households	31.08	Portnoy et al [18] Colombini et al [13]	Field study in Burkina Faso during the epidemic season (2006–2007) Costs includes transport, foods, costs for visitors, phone calls to the family, personal hygiene items Prorata of DMCs of Portnoy et al [18] as per the share between DNMCs and DMCs in Colombini et al [13]
Indirect costs for households	129.55	World Bank (GDP per capita); Colombini [13] (duration of inactivity)	Hypothesis (conservative): Duration of professional inactivity due to the illness = 21 days No. of persons impeded to work in the households = 1 Sequelae impact is not included here Formula of calculation: No. of days of inactivity × GDP/capita/day
Reactive vaccination campaign			
Vaccination			
Campaigns	263.25	Colombini et al [14]	Field study in Burkina Faso during the epidemic season (2006–2007). Includes meningococcal polysaccharide A/C vaccines and injection supplies; per diems and allowances for human resources; planning, training, social mobilization, monitoring, supervision and assessment of the immunization campaigns; management of cases of AEFIs; waste management; planning of overall surveillance and response activities (preepidemics)
Surveillance and other support activities	14.89	Colombini et al [14]	Field study in Burkina Faso during the epidemic season (2006–2007) Includes training, social communication on meningitis, investigation of suspected cases, laboratory case conﬁrmation and etiologic identiﬁcation, supervision, coordination of actors for surveillance and response activities
Preventive vaccination campaign			
Vaccination			
Delivery	Total costs	Gavi commitments and disbursements; countries application; campaigns evaluation report	Includes per diems and allowances for human resources; planning, training, social mobilization, monitoring, supervision and assessment of the immunization campaigns; surveillance; waste management; coordination, and partnership
Vaccine and injection material	Total costs	Gavi	Includes doses of MenAfriVac and injection supplies
Routine 1 dose, at 9 mo of age			
Vaccination	Mean Cost per Dose		
Delivery	0.28	cMYP Burkina Faso 2011–2015	Includes service delivery, advocacy and communication, monitoring and disease surveillance, program management. For comparison purpose, it does not include shared and capital costs (buildings, salaries of personnel, vehicles, and cold chain equipment)
Vaccine and injection material	0.90	MVP	Fully loaded price—includes doses of MenAfriVac, injection supplies and freight. Average price on the projection period (2015–2035)
Catch-up campaign			
Vaccination	Mean Cost per Dose		
Delivery	0.24	Current study	Hypothesis: The average delivery cost per dose for catch-up campaigns is the same as in the 2010 preventive campaigns
Vaccine and injection material	0.73	MVP	Fully loaded price—includes doses of MenAfriVac, injection supplies, and freight. Price as of 2015

Abbreviations: AEFI, adverse events following immunization; cMYP, comprehensive multiyear plan; DMC, direct medical cost; DNMC, direct nonmedical cost; GDP, gross domestic product; MVP, Meningitis Vaccine Project.

^a^ Value in 2012 US dollars.

The number of doses of vaccines required is based on the target population, the expected coverage rate, and wastage rates of each vaccination strategy (Table 3).

**Table 3.** Target Population, Coverage Rate, Wastage Factor, and Buffer Stock for Routine Vaccination and Preventive and Catch-up Campaigns Against Group A *Neisseria meningitidis*

Parameters	Routine Vaccination (2015–2035)	Catch-up Campaign (2015)	Preventive Campaigns (2010)
Total target population^a^	15 795 911	2 431 328	11 023 447
Coverage rate	80%	100%	100%^b^
Total effective target population^c^	12 212 780	2 995 304	11 023 447
Wastage factor	1.67	1.15	1.15
Buffer stock	25%	0%	0%
Total No. of vaccine doses^d^	20 643 066	2 796 027	12 205 400^e^

^a^ For the preventive campaigns, the target population is the one of 2010 only as the campaign is implemented only once during the total period.

^b^ Rate based on administrative coverage, used for the calculation of costs. A lower rate of 95%, based on a coverage survey estimate, is used for assessment of the epidemiological impact.

^c^ Routine effective target population = surviving infant population × rollout factor (accounting for partial year introduction in the first year) × coverage. Campaign effective target population = target population (assuming a plan to cover 100% of the target population).

^d^ Number of doses = [effective target population × wastage factor] + buffer. Buffer applies to routine only; for year x = 0.25 × [doses year x – Doses year x – 1] if this difference is positive; otherwise buffer = 0.

^e^ Source: World Health Organization. Summary report on the 2 phase meningitis vaccination campaign in Burkina Faso, January 2011.

#### Household Costs

Two types of costs for households are taken into account: DNMCs and ICs.

DNMCs are nonmedical costs incurred by the patient and/or the family carer because of the illness episode. For instance, in Burkina Faso, hospitals do not provide certain services (meals, hygiene) and there is hardly any transport or ambulance service. Consequently, households have to bear these transport costs, even for seriously ill patients. The family carers also have to pay for food for the patient and themselves, and buy soap and other personal hygiene items. Additionally, DNMCs include phone calls to family and costs for visitors (Table 2). This DNMC is calculated by multiplying Portnoy et al's DMC [18] by the ratio of DNMC and DMC in Burkina Faso from Colombini et al's original data (*r* = 0.61) [13].

ICs are estimated as the loss of income due to a temporary work interruption, calculated by multiplying the average number of days of illness (from [13]) by the daily per capita GDP (from the World Bank [20]). We considered that only 1 adult person per case was affected by work interruption: either the patient, if the patient is an adult, or 1 adult caregiver if the patient is a child.

### Savings and Economic Impact

The economic impact is estimated from the costs saved by both households and the health system. Savings are calculated as the difference between the costs of the baseline strategy (reactive strategy) and each alternative strategy over the same period of time; if the differential is positive, the alternative strategy is cost saving. The savings are subdivided by cost category (case management, vaccination, DNMCs, ICs).

## RESULTS

### Impact on Disease Burden

The number of cases of MenA expected in Burkina Faso varies from one strategy to the other (more detail is given in [12]). In the absence of preventive vaccination, 122 466 cases are predicted between 2015 and 2035. In contrast, the most effective combination strategy predicts only 3066 cases over the same period (Table 4). The 3 alternative strategies considerably reduce the number of cases of MenA, preventing at least 100 000 cases compared with the reactive strategy.

**Table 4.** Costs of Different Strategies of Vaccination Against Group A *Neisseria meningitidis*, Burkina Faso, 2010–2035

Strategy	No. of Cases of MenA	Health System			Households			Total
		Costs Case Management	Costs Vaccination	Subtotal	Direct Nonmedical Costs	Indirect Costs	Subtotal	
Reactive strategy								
2010–2014	20 453	1 037 575	5 688 847	6 726 422	635 669	2 649 616	3 285 284	10 011 706
2015–2035	122 466	6 212 720	34 063 283	40 276 002	3 806 212	15 865 184	19 671 396	59 947 398
1. Preventive strategy								
2010–2014	0	0	9 713 805	9 713 805	0	0	0	9 713 805
2. Routine strategy								
2015–2035	14 776	749 577	24 282 338	25 031 915	459 227	1 914 166	2 373 393	27 405 308
3. Combination strategy								
2015–2035	3066	155 550	27 000 288	27 155 838	95 298	397 223	492 520	27 648 358

Data are presented as US dollars unless otherwise specified.

Abbreviation: MenA, *Neisseria meningitidis* group A.

### Impact on Costs

The reactive strategy led to higher total costs both for the health system and for households, regardless of the comparison strategy (Table 4).

#### Total Undiscounted Costs

From 2010 to 2014, the costs of the preventive and the reactive strategy are almost the same, with 9.7 and 10.0 million USD, respectively (3.1% difference). However, the structure of the costs are very different: the cost of the preventive campaigns is 1.7 times that of the reactive campaigns, but there are no costs associated with cases under the preventive strategy as no MenA cases occurred during this time period.

Between 2015 and 2035, the cost of the reactive strategy is calculated as 59.9 million USD. By comparison, from 2015 to 2035 the routine and the combination strategies would cost a total of 27.4 to 24.6 million USD, respectively. De facto, the total cost for the reactive strategy is 2.2 times higher than the routine and the combination strategies. This is explained both by a higher number of cases of MenA, resulting in higher costs of case management and of DNMC and IC for households, and the higher costs of vaccination. The costs of the routine and the combination strategies are very similar due both to the low costs of the catch-up campaign in 2015 and to the lowest incidence of MenA under the combination strategy yielding lower case management costs, DNMCs, and ICs.

Within each strategy, total vaccination costs are higher than those linked to care of cases (case management, DNMCs, and ICs), due to vaccination leading to a decreased number of cases—and thus to a reduction of the costs linked to illness.

#### Costs for the Health System

A huge decrease in the costs of case management is observed for all preventive strategies compared with the reactive strategy, as many fewer cases are predicted to occur (Table 4). With routine and combination strategies, the costs of case management are only 2.7% and 0.6%, respectively, compared with 10.4% for the reactive strategy between 2015 and 2035.

Between 2010 and 2014, the health system costs associated with vaccination were higher for the preventive strategy, as all 1- to 29-year-olds in Burkina Faso were targeted nationwide, with costs concentrated in one single month in a single year (December 2010). By contrast, reactive campaigns selectively target districts experiencing epidemics and not the national population.

Between 2015 and 2035, vaccination costs of the reactive strategy are 1.4 to 1.3 times higher than those of the routine and the combination strategies, respectively (Table 4). The average vaccination cost per vaccinee is higher for routine vaccination than for preventive campaigns, with 1.99 USD and 0.88 USD respectively.

#### Costs for Households

The total cost for households is directly linked to the burden of disease: the fewer the cases, the lower the costs. All of the alternative strategies are less costly than the reactive strategy (Table 4), with the combination strategy resulting in the lowest household costs. ICs, which represent here the loss of earnings for the family during the acute illness episode and the recovery, represent the main costs to households (81%).

### Net Savings for Both the Health System and the Households

**Figure 1.**
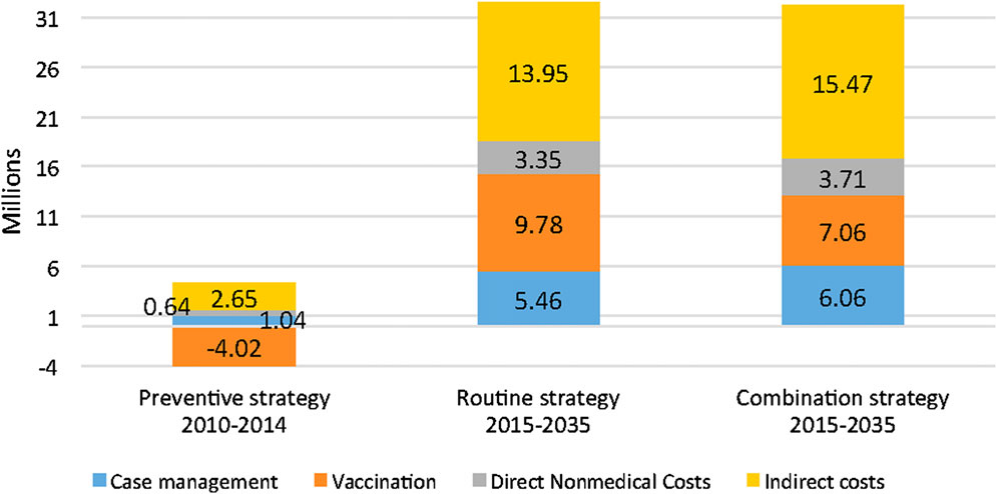
Savings (in millions of US dollars) of the 3 strategies, Burkina Faso, 2010–2035.

All the alternative strategies save money compared with the reactive strategy (Table 5, Figure 1). In total, the savings for the routine and the combination strategy are similar and amount to 32.5 and 32.3 million USD between 2015 and 2035, respectively. Savings are higher overall for the households than for the health system.

**Table 5.** Cost Savings of Various Strategies of Vaccination Against Group A Meningococcus, Burkina Faso, 2010–2035

Strategy	No. of Cases Averted	Health System (USD)			Households Savings (USD)			Total Savings (USD)
		Case Management	Vaccination	Subtotal	Direct Nonmedical Costs	Indirect Costs	Subtotal	
1. Preventive strategy, 2010–2014	20 453	1 037 575	−4 024 958	−2 987 383	635 669	2 649 616	3 285 284	297 901
2. Routine strategy, 2015–2035	107 690	5 463 143	9 780 945	15 244 088	3 346 985	13 951 018	17 298 003	32 542 090
3. Combination strategy, 2015–2035	119 400	6 057 170	7 062 995	13 120 165	3 710 914	15 467 961	19 178 875	32 299 040

Abbreviation: USD, US dollars.

Between 2010 and 2014, the savings are much lower (about 300 000 USD); there are no cases of MenA thanks to the preventive campaign, but this mass campaign was costly in terms of vaccination costs.

Ultimately, each dollar invested in routine immunization generates savings of an additional 1.3 USD, and each dollar invested in the combination strategy saves 1.2 USD.

### Sensitivity Analysis: Impact of Discounting

**Figure 2.**
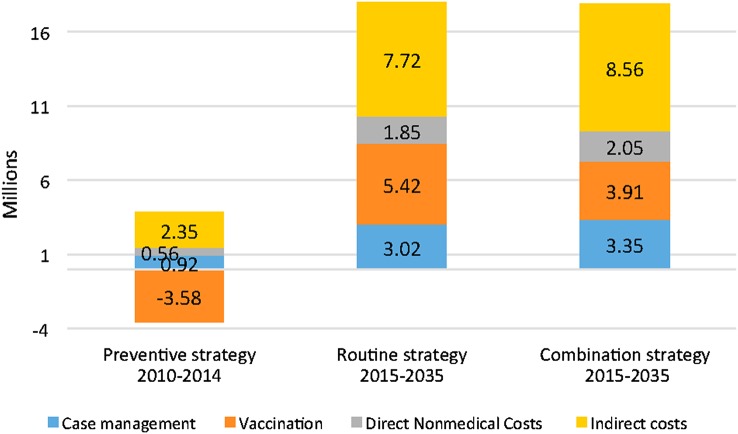
Discounted savings (in millions of US dollars) of the 3 strategies, Burkina Faso, 2010–2035. Savings are calculated based on a discounting of costs at a rate of 3%, but no discounting of cases (discount rate = 0%).

If discounting the costs at 3%, all the alternative strategies are still cost-saving both for the health system and for the households. However, the savings are lower than in the undiscounted scenario and amount to 17.8–18 million USD for the combination and the routine strategies, respectively (Figure 2). The only exception is again for vaccination, strictly speaking, under the preventive strategy between 2010 and 2014.

**Figure 3.**
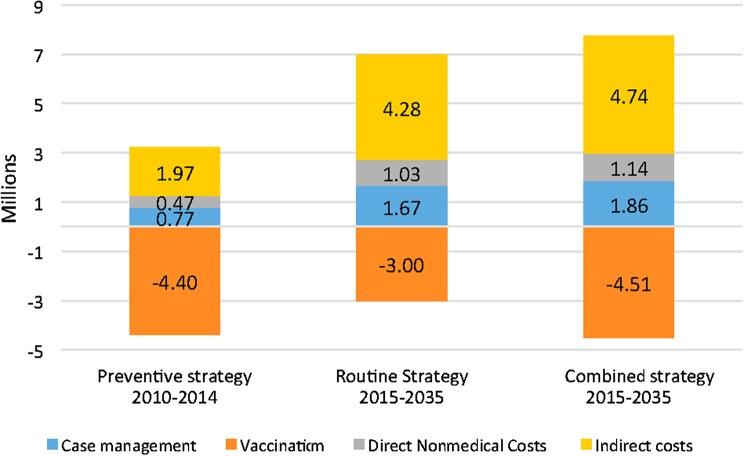
Discounted savings (in millions of US dollars) of the 3 strategies, Burkina Faso, 2010–2035. Savings relied on discounted costs and discounted cases at a discount rate of 3%.

When discounting both cases and costs at 3%, the preventive strategy costs 1.2 million USD more than the reactive strategy between 2010 and 2014. The long-term strategies remain cost-saving, with savings amounting to a maximum of 3.9 million USD for the routine strategy between 2015 and 2035. However, in this scenario vaccination costs are higher for each of the alternative strategies than for the reactive strategies (Figure 3).

## DISCUSSION

We find that the introduction and sustained use of MenAfriVac to prevent MenA has a substantial positive economic impact in Burkina Faso. Each of the preventive strategies considered generates considerable savings compared to the reactive strategy using a polysaccharide vaccine between 2015 and 2035, both with and without discounting costs at 3%. Indeed, each dollar invested in the combination strategy recommended by SAGE saves an additional 1.2 USD; in total, up to 32.3 million USD can be saved. Savings to the health system are accrued by lower costs of case management, and in the long term, also through reduced vaccination costs. By preventing MenA, MenAfriVac will reduce the economic burden on households and save thousands of households from catastrophic expenditures and pauperization [11]. Meningitis prevention is also likely to raise population well-being and development capacity including through the improvement of child health [21–24]. The severe disruption to the health system and communities from meningitis epidemics will be avoided [14, 25].

Our methodological choices were conservative and, thus, we may have underestimated the economic benefits of MenAfriVac. The costs to households may be underestimated for several reasons. We assumed that costs of case management are entirely supported by the health system; however, although care is supposed to be free of charge during an epidemic, an earlier study in Burkina Faso [13] estimated that 96% of households paid for all or part of the care. In addition, 34% of households report self-medication before going to a health center or a hospital, and 23% seek traditional care. The ICs were based on the assumption that only 1 person was prevented from working. In reality, this can only be true when the patient is a child, and although most cases occur in children, adults are still affected by MenA [26]. Household costs do not include either the direct or ICs associated with sequelae, which affect 9.5% of survivors [27], and may be severe and costly [13, 28]. Moreover, recent experimental economic studies propose widening the scope of ICs by also including monetary estimates of interruptions in education schooling and the impact on cognitive development [21–23]. Last, the focus here is on the economic impact on households and costs and savings for the health system. A later study will also include the macroeconomic impact and the financial risk protection of households [29].

Our estimates of the epidemiological impact of MenAfriVac from 2015 through 2035 are based on a transmission dynamic model of MenA [16]. Different MenAfriVac coverage estimates are used for the transmission model and the economic costs. For the 2010 campaign, costs are based on administrative coverage, whereas epidemiological impact uses a lower coverage survey estimate [3]; the overall effect is conservative. The transmission model predicts a national incidence and does not predict the occurrence of local epidemics that would trigger a reactive vaccination response. We have estimated the costs of reactive vaccination on a per-case basis. However, the costs of reactive vaccination could be lower if the geographical distribution of MenA cases was such that not all occur in districts that reach the epidemic response threshold. To address this, we used an alternative method of estimating costs of reactive vaccination, based on the number of district-level epidemic response campaigns conducted in Burkina Faso in the pre-MenAfriVac era and assuming a major epidemic every 10 years, and estimated costs to be around 10% higher. A further consideration is that we have not estimated the cases prevented through a reactive strategy. The effectiveness of reactive vaccination has not been systematically reviewed and critically depends on the speed at which reactive vaccination can be implemented [12]. However, it is not thought to be a highly effective strategy, hence the development and introduction of MenAfriVac. In addition, we are using an estimate of disease incidence that is typical for a meningitis belt country, although not necessarily a high-incidence country such as Burkina Faso. On balance, we therefore conclude that our estimates of the costs of reactive vaccination are conservative.

The introduction of MenAfriVac across the African meningitis belt has dramatically reduced the burden of MenA disease. With appropriate long-term immunization strategies as recommended by SAGE, this remarkable success promises to continue. The economic impact of MenAfriVac is illustrated here for Burkina Faso, and adds to the evidence on the remarkable public health success of this vaccine.
